# Child and Adolescent Mental Health Misinformation on TikTok: Cross-Sectional Mixed Methods Study

**DOI:** 10.2196/105698

**Published:** 2026-10-15

**Authors:** Constanza Testart Aguirre, Drigissa-Andrada Ilies, Alexandre Hudon

**Affiliations:** 1 Department of Psychiatry and Addictology Faculty of Medicine Université de Montréal Montreal, QC Canada; 2 Hôpital Rivière-des-Prairies Montreal, QC Canada; 3 Institut universitaire en santé mentale de Montréal Montreal, QC Canada; 4 Centre de recherche de l'Institut universitaire en santé mentale de Montréal Montreal, QC Canada

**Keywords:** TikTok, social media, misinformation, disinformation, mental health, child and adolescent mental health, psychoeducation, digital health, machine learning, content analysis

## Abstract

**Background:**

TikTok is an important source of mental health information for children, adolescents, and young adults, but the accuracy of psychoeducational content remains uncertain. Misleading information may be particularly consequential for younger users, who may have limited digital and health literacy.

**Objective:**

This study aimed to characterize misinformation and disinformation in TikTok content related to child and adolescent mental health, identify mental health topics disproportionately represented in misleading content, examine whether platform-observable video characteristics could discriminate videos containing misinformation, and explore how viewers responded to this content.

**Methods:**

We conducted a cross-sectional mixed methods secondary analysis of 122 child and adolescent mental health videos identified from a previously collected dataset of 1000 publicly available TikTok videos. Videos were independently coded for content, intent, authenticity, clinical topic, presenter characteristics, and engagement. An L2-penalized logistic regression using only platform-observable characteristics was evaluated using stratified 5-fold cross-validation; 95% CIs for predictive metrics were estimated using 5000 stratified bootstrap resamples of out-of-fold predictions. Disinformation was analyzed descriptively because only 2 cases were identified. An exploratory thematic analysis was conducted on 116 selected viewer comments.

**Results:**

Misinformation was identified in 21 of 122 (17.2%; 95% CI 11.5%-24.9%) videos, whereas disinformation occurred in 2 of 122 (1.6%; 95% CI 0.5%-5.8%). Misinformation was most frequent among videos addressing autism spectrum disorders (11/27, 40.7%), impulse control disorders (1/3, 33.3%), and mood disorders (4/13, 30.8%). Professional credentials were not disclosed in 63 of 122 (51.6%) videos, and only 9 of 122 (7.4%) referenced an external scientific source. The observable-feature model demonstrated modest discrimination, with an area under the receiver operating characteristic curve of 0.70 (95% CI 0.56-0.82), balanced accuracy of 0.69 (95% CI 0.58-0.79), recall of 0.67 (95% CI 0.48-0.86), and *F*_1_-score of 0.44 (95% CI 0.32-0.56). Qualitative analysis identified 5 recurring patterns in viewer responses: sharing and validation of lived experiences, discussion of diagnosis and misdiagnosis, frustration with mental health care, peer support and encouragement, and exchanges about treatment and medication experiences.

**Conclusions:**

A substantial minority of the analyzed TikTok videos contained misinformation, with particularly high proportions among some neurodevelopmental and mood-related topics. Platform-observable characteristics provided only modest discrimination of misinformation, underscoring the difficulty of identifying misleading mental health content from surface-level features alone. Viewer comments further showed that TikTok mental health content is embedded within broader processes of self-interpretation, diagnostic discussion, peer validation, and treatment-related exchange. These findings support the need for developmentally appropriate digital health literacy initiatives and accurate, transparently sourced psychoeducational content for young social media users.

## Introduction

### Social Media Use and Youth Mental Health

Social media (SM) use has grown immensely worldwide, with 5.22 billion active SM user identities in October 2024, representing 63.8% of the global population [1]. Among youths, SM adoption is now nearly universal, reaching up to 95% of adolescents, while approximately 40% of children aged 8 to 12 years are also active online [2,3]. The time spent online by US adolescents doubled in the last 10 years, nearly half of adolescents being online almost constantly versus only a quarter a decade ago [4]. Average American adolescents and adults spend about 4 to 6 hours and 1 hour per day on SM, respectively [5,6]. TikTok is the most popular SM network among young users worldwide and the second most popular platform among US adolescents after YouTube with 63% versus 90% of users, respectively [7]. About 6-in-10 teenagers visit TikTok daily. This includes 16% who report being on it almost constantly [4].

During the past 15 years, the increased SM use has amassed growing attention in connection with a significant increase in mental health issues among youths observed globally [8]. Multiple studies have found a negative relationship between SM use and mental health problems, with a heightened risk for children and adolescents [6,9]. A systematic review of the studies exploring the relationship between TikTok use and adolescent mental health showed an overall negative impact of TikTok use on the mental health of adolescents, notably lower life satisfaction, increased depressive symptoms, higher levels of anger and loneliness, risk of problematic TikTok use, increased body image concerns and self-esteem issues, as well as the rapid spreading of functional tic-toc like behaviors [8]. Higher SM use was associated with low self-esteem and increased depression and anxiety symptoms and depression rates in young adults and adolescents [10,11]. In addition, prolonged SM was linked to self-injurious behavior and suicidality in adolescents [6,12].

### Mental Health Misinformation on SM

SM platforms, including TikTok, have become an important source of mental health information for both pediatric and adult populations [13,14]. While they offer opportunities for social connection and sharing experiences and strategies for health and wellness, SM also represent a space for the dissemination of erroneous or false information, which are serious issues that considerably impact public trust and health [15].

Misinformation refers to false or inaccurate information shared without the intent to deceive, whereas disinformation is intentionally false information disseminated to mislead audiences. The rapid growth of SM has amplified the spread of both, allowing inaccurate health information to reach millions of users within hours through algorithm-driven recommendation systems. Health misinformation has been associated with increased treatment hesitancy, reduced trust in health care professionals and scientific institutions, and the adoption of harmful health behaviors, making it a growing public health concern. Mental health is particularly vulnerable because psychiatric disorders often involve subjective symptoms and complex diagnostic criteria that can easily be oversimplified or misrepresented online. Consequently, misleading content may perpetuate stigma, promote misconceptions regarding the causes and treatment of mental disorders, and discourage individuals from seeking evidence-based care [16-18].

Children and adolescents may be especially susceptible to mental health misinformation because they are among the most active users of SM while often possessing lower levels of digital and health literacy than adults. During adolescence, ongoing cognitive and socioemotional development may further reduce the ability to critically evaluate online information, increasing vulnerability to misleading or sensationalized mental health content. Parents, caregivers, and educators may also unknowingly disseminate inaccurate information, further amplifying its influence on young people [19].

Unsurprisingly, mental health misinformation is widespread across SM platforms, particularly TikTok, where short-form videos frequently accumulate millions of views. Systematic reviews consistently report that the quality of mental health information available on SM is highly variable, with substantial proportions of content containing inaccurate, misleading, or overgeneralized claims [20]. Neurodevelopmental disorders such as autism spectrum disorder (ASD) and attention-deficit/hyperactivity disorder (ADHD) have been studied extensively because they are among the platform’s most viewed mental health topics. Studies evaluating highly viewed TikTok videos found that only a minority provided accurate psychoeducational information, whereas many contained misleading descriptions of symptoms or diagnostic criteria [21,22]. Such portrayals may encourage the pathologization of normative experiences, reinforce self-diagnosis, and delay appropriate professional assessment. Although SM can improve mental health awareness and reduce stigma, the widespread dissemination of inaccurate content highlights the need for systematic evaluation of the quality and potential impact of mental health information shared on contemporary SM platforms.

### Addressing the Quality of Mental Health Content on TikTok

Griffiths et al [23] evaluated the quality of the web-based information on treatment of depression and highlighted that popular websites often omitted key information about the disorder and treatments and made treatment recommendations that contradicted established guidelines without providing supporting evidence. Similarly, Turuba et al [24] aimed to understand the nature of depression-related content and its potential impact on viewers. One of the key findings showed that a significant portion of the content contained misinformation or lacked evidence-based information about depression. They emphasized the need for educational campaigns to improve users’ understanding of depression and promote the dissemination of accurate information, as well as interventions aimed at reducing misinformation on the platform and strengthening users’ ability to distinguish anecdotal claims from evidence-based information.

Despite the risks associated with mental health misinformation or disinformation on SM platforms and world-wide popularity of SM, which shapes public perception about mental health and neurodevelopmental conditions, studies focused on improving the quality on mental health content are scarce [25]. A recent 2024 study emphasized that mental health content coaching sessions for creators could improve their content quality and the reach out of their accounts [26]. While these results are interesting, they do not provide concrete and summarized recommendations on how to build good-quality short-form content on TikTok. Moreover, there are no recommendations for children, adolescent, or young adult users on how to identify a good-quality video versus misinformation or disinformation.

### Objectives and Hypotheses

This exploratory study aimed to characterize misinformation and disinformation in TikTok content addressing child and adolescent mental health and to identify the mental health topics most frequently represented among videos containing misleading or false information. A further objective was to examine whether platform-observable characteristics, including engagement metrics, video duration, hashtag use, declared professional credentials, and mental health topic, could discriminate videos containing misinformation. The study also explored how viewers responded to this content through qualitative analysis of selected comments and sought to generate evidence-informed recommendations for the development of high-quality psychoeducational short-form content for children, adolescents, and young adults.

Based on previous literature, we hypothesized that misinformation would be present in a meaningful proportion of child and adolescent mental health content on TikTok and that neurodevelopmental conditions, particularly ASD and ADHD, as well as mood disorders, would be disproportionately represented among videos containing misinformation. Because the predictive component was exploratory, no directional hypotheses were specified for individual platform-observable characteristics. Similarly, the qualitative analysis was inductive, and no a priori hypotheses were formulated regarding viewer-comment themes.

## Methods

### Study Design and Overview

A cross-sectional observational content analysis of short-form videos posted on TikTok that addressed child or adolescent mental health topics was carried out. The study followed established guidance for social media infodemiology, used a structured coding framework derived from Aïmeur et al [27], and incorporated descriptive statistics and an exploratory penalized logistic regression model to examine platform-observable characteristics associated with misinformation.

### Participants and Recruitment

This study was a secondary analysis of an existing dataset of 1000 publicly available TikTok videos previously collected and analyzed by Hudon et al [28]. No new TikTok videos were collected specifically for this study. As described in the parent study, candidate videos were identified using TikTok’s algorithmically curated “For You” feed, informed by popular mental health hashtags and search trends. Newly created neutral accounts without prior engagement histories were used during the original data collection to reduce personalization of the recommendation algorithm.

For this study, all 1000 videos in the parent dataset were screened for relevance to child and adolescent mental health. Videos were retained when they specifically addressed psychological or psychiatric conditions, mental health or well-being, or diagnostic or treatment issues involving children or adolescents. The parent dataset had already been restricted to psychoeducational or intended-to-be-psychoeducational content available in English, French, or Spanish, excluding multipart videos and content inaccessible without a TikTok account [28]. Application of the additional child and adolescent mental health criterion resulted in a final analytic sample of 122 videos; 878 videos were excluded because they did not meet this additional criterion.

Although French was an eligible language in the parent dataset, no French-language videos met the additional eligibility criterion for the present analysis. The resulting sample therefore comprised 67 English-language and 55 Spanish-language videos.

Eight coders (clinical psychologists, psychiatrists, and research assistants) completed a 1-hour calibration workshop with written guidance and practice exercises. Interrater reliability was assessed on the 50-video calibration subset using Krippendorff α for nominal data. Separate coefficients were calculated for content, intent, and authenticity from the preconsensus ratings. Following calibration, all 122 videos were independently double-coded, and disagreements were resolved by consensus. Interrater reliability was high across the principal coding dimensions, with Krippendorff α values of 0.88 for content, 0.91 for intent, and 0.84 for authenticity.

### Data Collection

A dataset was collaboratively constructed by all the authors, identifying key elements from 3 different categories for each analyzed video: video characteristics, quality of the information extracted, and clinical elements related to mental health. The data collected in this study are presented in Textbox 1. Weighted numbers for likes, comments, shares, and favorites were obtained by dividing their total amount by the number of days elapsed since the upload date.

**Categories and variables collected for each identified TikTok video (adapted from Hudon et al [28], which is published under Creative Commons Attribution 4.0 International License [29]).**

**Video characteristics**

- Video upload date
- Country
- Professional title of the presenter (self-reported title)
- Language
- Length (in minutes)
- Weighted number of likes
- Weighted number of comments
- Weighted number of shares
- Weighted number of favorites
- Target audience (general or specific audience)
- Hashtags used

**Quality of the information**

- Content (opinion-based, fact-based, and mixed)
- Intent (disinformation, misinformation, clickbait, satire, and other).
- Authenticity (rumor, propaganda, hoax, conspiracy, framing, reference-based, and other)

**Clinical mental health data**

- Topic (main topic)
- Reference used

The criteria used to assess information quality were adapted from the framework developed by Aïmeur et al [27], who proposed formal definitions of content, intent, and authenticity within the context of fake news. In this study, content was categorized as opinion-based, fact-based, or mixed. The intent dimension classified videos as misinformation, disinformation, clickbait, satire, or other. Misinformation was operationalized as false, inaccurate, misleading, or scientifically unsupported content for which there was no observable evidence of deliberate deceptive intent. Disinformation was operationalized as false or misleading content for which observable elements of the video or its context suggested deliberate deceptive intent. Because creator intent cannot be directly verified from publicly available SM content, these classifications reflect coder inference based on observable material rather than direct knowledge of the creator’s intentions. Clickbait referred to content primarily designed to attract attention or promote a product or service, satire to humorous or parodic presentations, and other to informative, educational, experiential, or otherwise relevant content that did not meet criteria for misinformation, disinformation, clickbait, or satire. Authenticity referred to the nature of the information source and included rumor, propaganda, hoax, conspiracy, framing, reference-based, and other categories.

### Data Analysis

Descriptive statistics, including frequencies, proportions, and measures of central tendency and dispersion, were calculated in Python (version 3.9; Python Software Foundation).

To explore whether platform-observable characteristics could discriminate videos containing misinformation, we fitted an L2-penalized logistic regression model. Predictor selection was restricted to characteristics that could be observed independently of the study’s information-quality coding: weighted likes, comments, favorites, and shares; video duration; number of hashtags; whether professional credentials were declared; and mental health topic. Coder-assigned content, intent-related variables other than the misinformation outcome, and authenticity classifications were excluded to avoid conceptual circularity. Engagement variables were log-transformed using log(1+*x*) because of their skewed distributions and, together with video duration and hashtag count, standardized before modeling. Mental health topic was one-hot encoded, with attention disorders as the reference category. Professional credential disclosure was represented as a binary variable. Class weights were inversely proportional to class frequencies. Model performance was evaluated using stratified 5-fold cross-validation, generating out-of-fold predictions for all 122 videos at a classification threshold of 0.50. Accuracy, precision, recall, *F*_1_-score, balanced accuracy, area under the receiver operating characteristic curve (AUROC), average precision, and the confusion matrix were reported and compared with a majority-class baseline. The 95% CIs were calculated using 5000 stratified bootstrap resamples of the out-of-fold predictions.

The qualitative component examined viewer comments rather than video transcripts. During data extraction, coders copied 1 viewer comment judged to be illustrative of the discussion associated with each video. Comment text was available for 116 of the 122 videos; 6 videos had no comment recorded in the analytic dataset. The 116 comments were analyzed using an inductive thematic approach informed by Braun and Clarke [30]. Two reviewers (CTA and AH) independently coded the comments to identify recurring patterns related to viewers’ responses to child and adolescent mental health content. Candidate themes were subsequently compared, discussed, refined, and organized into a final thematic framework. Because comments were not sampled exhaustively or according to a predefined engagement-based ranking, the qualitative analysis was exploratory and was intended to characterize recurring patterns rather than estimate the frequency or prevalence of themes among TikTok users.

### Ethical Considerations

The ethics committee of the Centre de recherche de l’Institut universitaire en santé mentale de Montréal was consulted to determine the need for ethics approval for this study’s objectives and methodology. According to the Canadian Tri-Council Policy Statement on Ethical Conduct for Research Involving Humans, no ethics board approval was required, as the TikTok videos analyzed belong to the public domain. All data have been deidentified.

## Results

### Overview

A total of 122 TikTok videos were analyzed. Slightly more than half were in English (67/122, 54.9%). Country of origin was not stated in most cases (83/122, 68%); the United States (17/122, 13.9%), Canada (6/122, 4.9%), and the United Kingdom (3/122, 2.5%) were the next most common. Presenters’ professional status was usually unclear (not specified: 63/122, 51.6%). When disclosed, the largest groups were psychologists (16/122, 13.1%), psychiatrists (11/122, 9%), and therapists (6/122, 4.9%). Engagement distributions were markedly right-skewed. Median weighted engagement was 29.5 (IQR 3.3-209.2) likes per day, 0.6 (IQR 0.1-5.0) comments per day, 5.9 (IQR 0.7-50.1) favorites per day, and 2.41 (IQR 0.4-32.8) shares per day. Corresponding mean values were 661.5 (SD 2212.6), 12.8 (SD 46.3), 81.0 (SD 244.2), and 73.0 (SD 299.5) per day, respectively. Distributions of mental health topics are presented in Figure 1. Language, geographic origin, and self-reported presenter credentials are presented in Table 1. Distribution of coder-assigned intent categories by mental health topic is found in Table 2.

**Figure 1:**
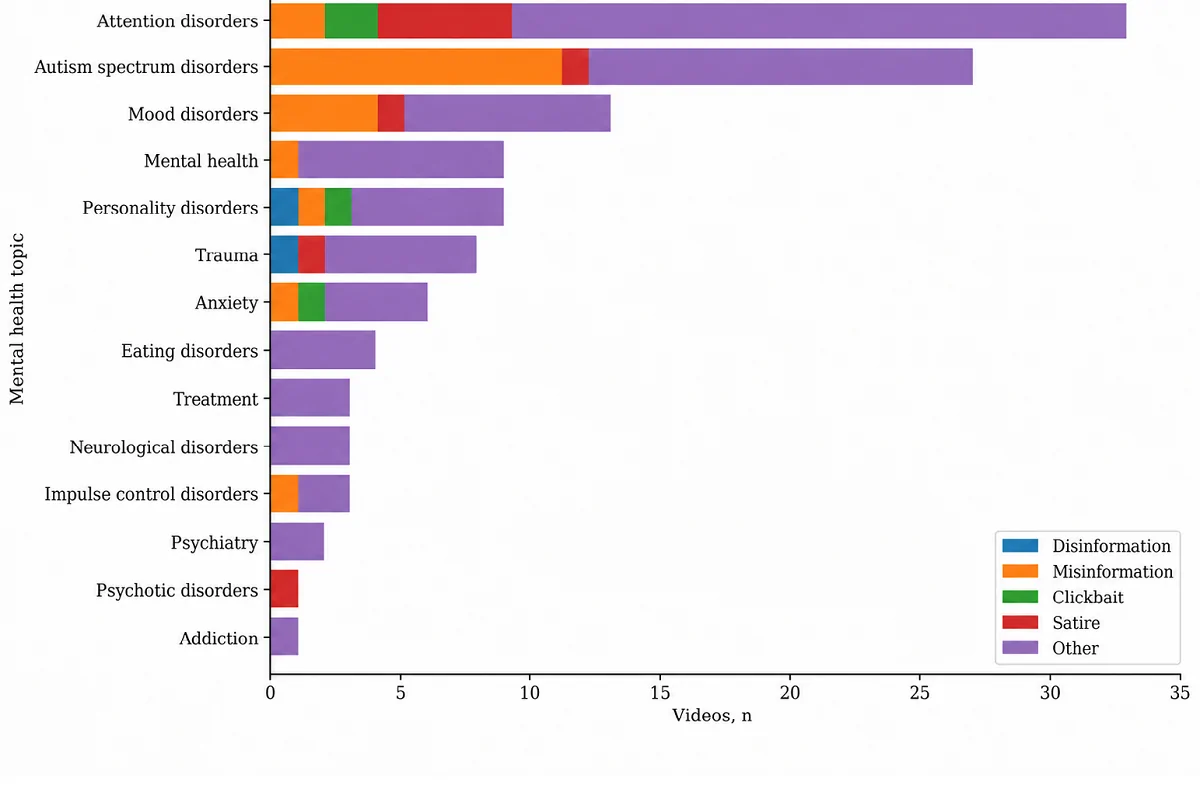
Distribution of intent categories across mental health topics in a cross-sectional secondary analysis of 122 publicly available TikTok videos addressing child and adolescent mental health. The online sample comprised 67 English-language and 55 Spanish-language videos. Horizontal stacked bars show the absolute number of videos within each mental health topic classified as disinformation, misinformation, clickbait, satire, or other; topic-specific denominators therefore vary.

**Table 1:** Language, geographic origin, and self-reported presenter credentials of 122 publicly available TikTok videos addressing child and adolescent mental health in a cross-sectional secondary content analysis^a^.

Variable		Values, n (%)
Language		
	English	67 (54.9)
	Spanish	55 (45.1)
Country		
	United States	17 (13.9)
	Canada	6 (4.9)
	United Kingdom	3 (2.5)
	Mexico	3 (2.5)
	Sweden	2 (1.6)
	Australia	2 (1.6)
	India	2 (1.6)
	Ecuador	2 (1.6)
	Peru	1 (0.8)
	Colombia	1 (0.8)
	Not specified	83 (68)
Presented by (professional title)		
	Psychologist	16 (13.1)
	Psychiatrist	11 (9)
	Therapist	6 (4.9)
	Medical doctor	3 (2.5)
	Advisor	3 (2.5)
	Neuropsychologist	3 (2.5)
	Guru	2 (1.6)
	Neurologist	2 (1.6)
	Doctor	2 (1.6)
	Coach	1 (0.8)
	Holistic psychologist, hypnotherapist	1 (0.8)
	Pediatrician	1 (0.8)
	Actor	1 (0.8)
	Holistic pediatrician	1 (0.8)
	Psychology student	1 (0.8)
	Counselor	1 (0.8)
	Plastic surgeon	1 (0.8)
	Podcaster	1 (0.8)
	School principal	1 (0.8)
	Hairstylist	1 (0.8)
	Not specified	63 (51.6)

^a^Videos were drawn from an international online sample; geographic origin was not specified for 83 of 122 (68%) videos.

**Table 2:** Distribution of coder-assigned intent categories by mental health topic among 122 publicly available TikTok videos addressing child and adolescent mental health in a cross-sectional secondary content analysis^a^.

Mental health topic	Total, n	Disinformation, n/N (%)	Misinformation, n/N (%)	Clickbait, n/N (%)	Satire, n/N (%)	Other, n/N (%)
Attention disorders	33	0/33 (0)	2/33 (6.1)	2/33 (6.1)	5/33 (15.2)	24/33 (72.7)
Autism spectrum disorders	27	0/27 (0)	11/27 (40.7)	0/27 (0)	1/27 (3.7)	15/27 (55.6)
Mood disorders	13	0/13 (0)	4/13 (30.8)	0/13 (0)	1/13 (7.7)	8/13 (61.5)
Mental health	9	0/9 (0)	1/9 (11.1)	0/9 (0)	0/9 (0)	8/9 (88.9)
Personality disorders	9	1/9 (11.1)	1/9 (11.1)	1/9 (11.1)	0/9 (0)	6/9 (66.7)
Trauma	8	1/8 (12.5)	0/8 (0)	0/8 (0)	1/8 (12.5)	6/8 (75)
Anxiety	6	0/6 (0)	1/6 (16.7)	1/6 (16.7)	0/6 (0)	4/6 (66.7)
Eating disorders	4	0/4 (0)	0/4 (0)	0/4 (0)	0/4 (0)	4/4 (100)
Treatment	3	0/3 (0)	0/3 (0)	0/3 (0)	0/3 (0)	3/3 (100)
Neurological disorders	3	0/3 (0)	0/3 (0)	0/3 (0)	0/3 (0)	3/3 (100)
Impulse control disorders	3	0/3 (0)	1/3 (33.3)	0/3 (0)	0/3 (0)	2/3 (66.7)
Psychiatry	2	0/2 (0)	0/2 (0)	0/2 (0)	0/2 (0)	2/2 (100)
Psychotic disorders	1	0/1 (0)	0/1 (0)	0/1 (0)	1/1 (100)	0/1 (0)
Addiction	1	0/1 (0)	0/1 (0)	0/1 (0)	0/1 (0)	1/1 (100)
Total	122	2/122 (1.6)	21/122 (17.2)	4/122 (3.3)	9/122 (7.4)	86/122 (70.5)

^a^Intent was classified as disinformation, misinformation, clickbait, satire, or other. Percentages within individual topic rows use the number of videos addressing that topic as the denominator. Percentages in the total row use n=122.

### Information Quality Profile

Analysis of the informational content revealed that nearly half of all videos (60/122, 49.2%) were primarily fact-based, presenting information consistent with known psychological or psychiatric frameworks. In contrast, 29 of 122 (23.8%) videos were coded as purely opinion, often drawing from personal anecdotes or subjective interpretations, while 33 of 122 (27.0%) fell into a mixed category, blending factual claims with individual perspectives.

With respect to intent, misinformation was present in 21 of 122 (17.2%) videos. In contrast, disinformation, or deliberately misleading content, was much rarer, occurring in only 2 of 122 (1.6%) videos. Additionally, 4 of 122 (3.3%) videos were identified as clickbait, marked by exaggerated titles or thumbnails intended to attract engagement. A further 9 of 122 (7.4%) were categorized as satire or parody, and the remaining 86 of 122 (70.5%) comprised content coded as “other,” typically including benign personal testimony or general mental health advice without overtly misleading claims.

The authenticity dimension revealed further concerns. Approximately 12 of 122 (9.8%) videos were classified as hoaxes, presenting clearly fabricated or implausible information. Rumors accounted for 5 of 122 (4.1%) videos, and propaganda (content promoting an ideological agenda under the guise of health information) represented 3 of 122 (2.5%). Only 9 of 122 (7.4%) videos made any reference to external scientific sources, suggesting a limited presence of evidence-based psychoeducational content on the platform.

The platform-observable feature model demonstrated modest discrimination of videos containing misinformation. Across cross-validated out-of-fold predictions, 14 of 21 misinformation videos were correctly identified, whereas 72 of 101 videos without misinformation were correctly classified, yielding 29 false-positive and 7 false-negative classifications. Accuracy was 0.70 (95% CI 0.6-0.8), precision 0.3 (95% CI 0.2-0.4), recall 0.7 (95% CI 0.5-0.9), *F*_1_-score 0.4 (95% CI 0.3-0.6), balanced accuracy 0.7 (95% CI 0.6-0.8), AUROC 0.70 (95% CI 0.6-0.8), and average precision 0.3 (95% CI 0.2-0.6). Although the majority-class baseline achieved higher raw accuracy (82.8%) by classifying every video as not containing misinformation, it had 0 sensitivity, and an *F*_1_-score of 0. These findings indicate that observable video characteristics provided some discrimination beyond the majority-class strategy, but overall predictive performance remained modest. Model’s performance metrics are presented in Table 3.

In the model fitted to the complete dataset, the direction of penalized coefficients varied across observable characteristics. Weighted shares showed a positive association with the modeled log-odds of misinformation, whereas weighted favorites, likes, and comments showed negative coefficients after simultaneous adjustment for the other variables. Declared professional credentials also showed a negative coefficient. Topic-related coefficients were heterogeneous, with ASD and mood disorder content showing the largest positive coefficients relative to attention disorders. Given the small number of misinformation cases and the exploratory nature of the model, these coefficients should not be interpreted as causal effects or stable risk factors. The top exploratory associations are presented in Figure 2.

**Table 3:** Cross-validated performance of an L2-penalized logistic regression model using platform-observable characteristics to discriminate misinformation among 122 publicly available TikTok videos addressing child and adolescent mental health^a^.

Model	TN^b^	FP^c^	FN^d^	TP^e^	Accuracy (95% CI)	Recall (95% CI)	Precision (95% CI)	*F*_1_-score (95% CI)	Balanced accuracy (95% CI)	AUROC^f^ (95% CI)	Average precision (95% CI)
Observable-feature penalized regression	72	29	7	14	0.7 (0.6-0.8)	0.7(0.5-0.9)	0.3 (0.2-0.4)	0.4 (0.3-0.6)	0.7 (0.6-0.8)	0.7 (0.6-0.8)	0.3(0.2-0.6)
Majority-class baseline	101	0	21	0	0.8 (0.7-0.0)	0 (0.00-0.2)	0 (0.0-0.0)	0 (0.0-0.0)	0.5 (0.5-0.5)	0.5 (0.5-0.5)	0.2 (0.1-0.3)

^a^Model performance was estimated using stratified 5-fold cross-validation with out-of-fold predictions and compared with a majority-class baseline.

^b^TN: true negative.

^c^FP: false positive.

^d^FN: false negative.

^e^TP: true positive.

^f^AUROC: area under the receiver operating characteristic curve.

**Figure 2:**
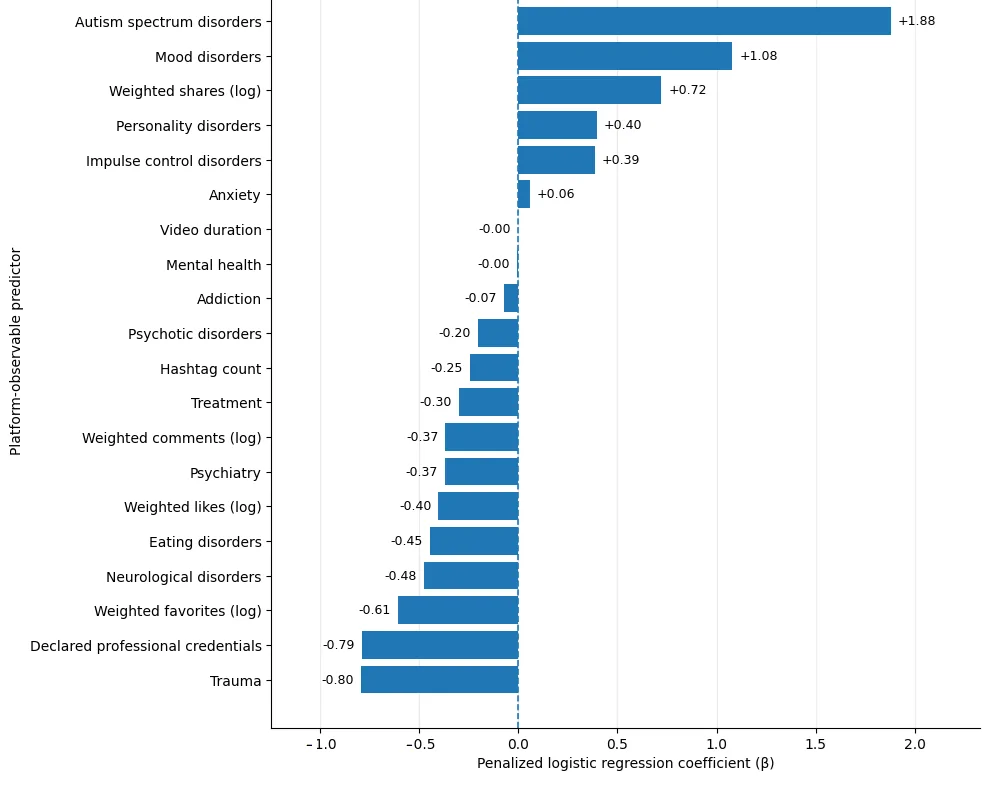
Exploratory associations between platform-observable video characteristics and misinformation in a cross-sectional secondary analysis of 122 publicly available TikTok videos addressing child and adolescent mental health. Bars represent coefficients from an L2-penalized logistic regression fitted to the misinformation outcome (21/122 videos). Continuous predictors were standardized before modeling; mental health topic coefficients are relative to attention disorders. Positive coefficients indicate higher modeled log-odds, and negative coefficients indicate lower modeled log-odds of misinformation after simultaneous adjustment for included variables. Coefficients are exploratory associations and should not be interpreted as causal effects or stable risk factors.

### Qualitative Themes From Selected Viewer Comments

#### Overview

A viewer comment was available for 116 of the 122 videos. Exploratory thematic analysis of these selected comments identified 5 recurring themes: personal experience and validation, diagnosis and misdiagnosis, frustration and criticism of mental health care, support and encouragement, and treatment and medication experiences. The flow diagram pertinent to the video selection as per the qualitative assessment portion of this study is presented in Figure 3.

**Figure 3:**
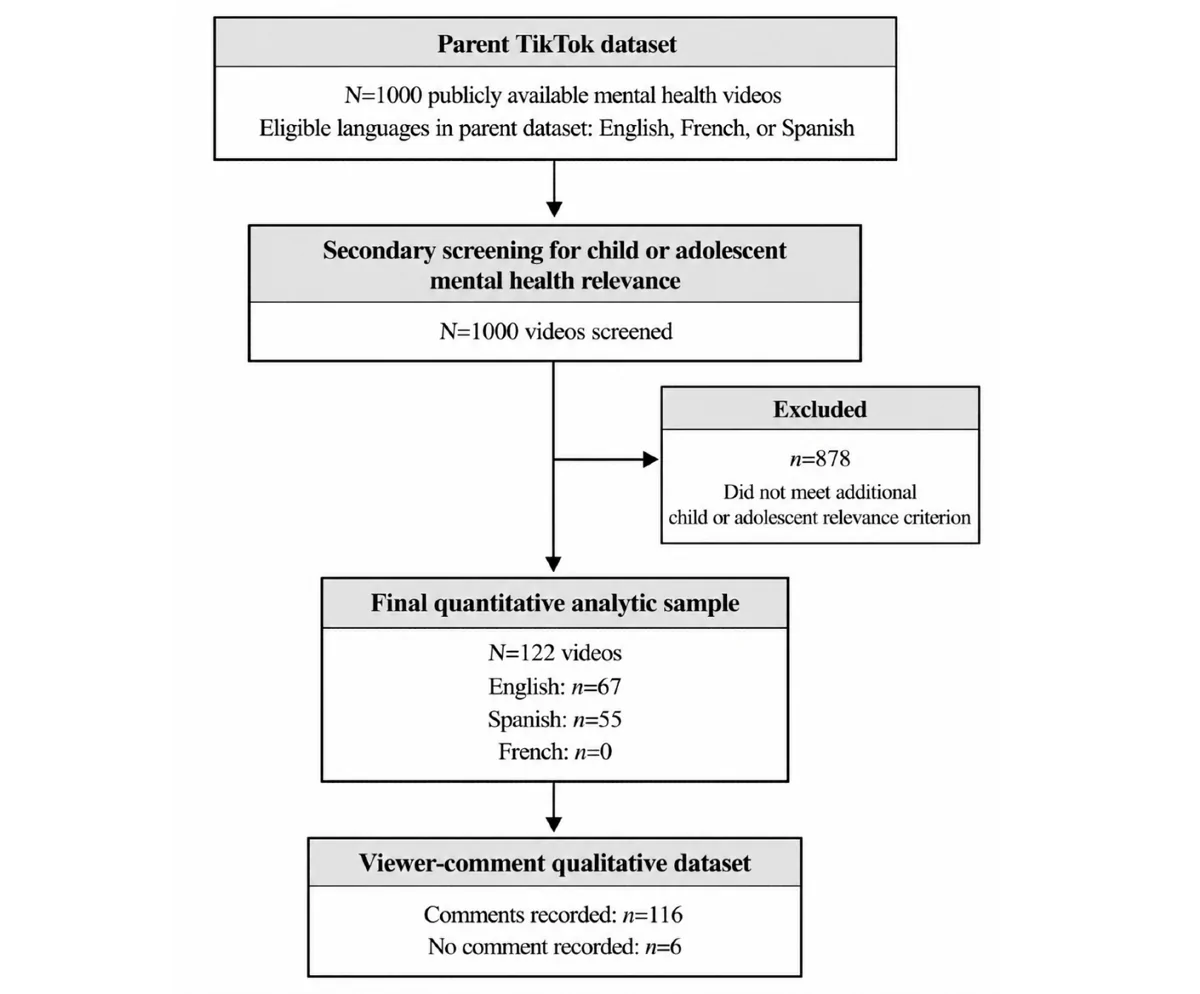
Flow of videos from the parent TikTok dataset to the current child and adolescent mental health analytic sample.

#### Personal Experience and Validation

Many users shared their lived experiences, often in response to content that resonated with their own challenges or those of their family members. These comments created a sense of shared understanding and community among viewers. Examples included statements such as “Exactly, I’ve been through this,” “This is my son as well,” and “I have tried Fluoxetine and Sertraline.” These contributions suggest that viewers frequently used the comment sections as spaces for affirmation, empathy, and storytelling.

#### Mental Health Diagnosis and Misdiagnosis

A notable portion of comments raised concerns about the accuracy or applicability of certain diagnoses. Users expressed skepticism about overdiagnosis or misinterpretation of normative behavior, especially in children. Illustrative comments included “Hmm could be a misdiagnosis for autism” and “These are signs of a typical toddler, not autism.” Such reflections point to public discourse grappling with the boundaries of diagnostic categories and a perceived tendency to medicalize everyday experiences.

#### Frustration and Criticism of Mental Health Care

Several users articulated dissatisfaction with the mental health system, as well as frustration with dismissive attitudes from clinicians, caregivers, or peers. These expressions highlighted gaps in care, misunderstandings within support networks, and broader systemic challenges. Representative quotes included “It’s so dismissive and frustrating” and “My mom: ‘It’s that damn phone.’”

#### Support and Encouragement

Despite critical voices, many comments were supportive in tone, offering encouragement and emotional solidarity. These included expressions of positivity such as “I love this” and belief-oriented affirmations like “Believe it’s because their energy is so negative.” Such responses suggest that viewers also use TikTok as a platform for emotional reinforcement and community-based coping.

#### Treatment and Medication Experiences

Finally, a number of comments reflected personal experiences with psychiatric treatments, particularly medication. Users shared both successes and frustrations, contributing to a discourse around pharmacotherapy. Examples, such as “I have tried Fluoxetine and Sertraline,” highlighted how individuals publicly engage in discussions about mental health interventions, offering peer-to-peer insight into medication effects.

## Discussion

### Principal Findings

This study offers one of the first comprehensive explorations of psychoeducational short-form content on TikTok related to child and adolescent mental health. Through a mixed methods design combining content analysis, exploratory analysis of viewer comments, and penalized logistic regression, we characterized misinformation and disinformation and examined whether platform-observable characteristics could discriminate videos containing misinformation.

### Comparison With Prior Work

From the 122 analyzed videos, a nonnegligible portion (17.2%) contained misinformation, among whom those discussing content about ASDs (11/27, 40.7%), impulse control disorders (1/3, 33.3%), and mood disorders (4/13, 30.8%) were the most represented. This is in line with the results of previous research on ASD-related TikTok content, which primarily addressed the reach and accuracy of informational videos, finding a prevalence of inaccuracies and overgeneralizations [21,31]. As an example, Aragon-Guevara et al [21] reported that among the most popular TikTok videos providing psychoeducation about autism, 41% were inaccurate and 32% contained overgeneralized misleading information. Similarly, Brown et al [32] described that from 100 videos about autism on TikTok, 41% were classified by psychiatrists and medical trainees as “misleading.”

Disinformation was identified in only 2 of 122 (1.6%) videos, comprising 1 of 8 (12.5%) trauma videos and 1 of 9 (11.1%) personality-disorder videos. Given the very small number of disinformation cases, no predictive model was fitted for this outcome. For misinformation, the revised L2-penalized logistic regression restricted to platform-observable characteristics demonstrated only modest discrimination (AUROC 0.70, 95% CI 0.56-0.82; balanced accuracy 0.69, 95% CI 0.58-0.80). Associations between individual observable features and misinformation were heterogeneous and should be interpreted as exploratory rather than as stable risk factors or causal effects. Qualitative analysis of viewer comments further contextualized these findings, revealing recurring patterns of personal storytelling, diagnostic uncertainty, frustration with mental health care, peer support, and treatment-related discussion. These findings suggest that misinformation cannot be reliably identified from surface-level platform characteristics alone and that its interpretation should also consider the broader social and conversational context in which mental health content is consumed.

While previous research has identified the spread of health misinformation during the COVID-19 pandemic and within online mental health forums, few studies have operationalized these conceptual dimensions with such granularity in a short-form video context [33,34]. Descriptively, only 9 of 122 (7.4%) videos referenced external scientific sources, highlighting the limited explicit sourcing of mental health information in this sample. Engagement-related characteristics showed heterogeneous associations in the exploratory model, and these findings should not be interpreted as evidence that virality causes or algorithmically amplifies misinformation. Previous studies have similarly raised concerns regarding the relationship between engagement and the quality of health information on SM [35]. The lack of external citations in over 90% of videos also echoes prior concerns regarding the evidence gap in user-generated mental health media [24]. Similarly, as Steiner-Hofbauer et al [25] showed, in a sample of TikTok autism-related videos, a higher public engagement in terms of views and comments with the videos classified as personal experience, with no evidence-based information.

Another salient theme concerns the perceived legitimacy of presenters. More than half of presenters did not declare professional credentials. In the exploratory penalized model, declared professional credentials had a negative coefficient, but given the limited sample and number of misinformation cases, this finding should not be interpreted as evidence of a protective effect. This finding resonates with the work of Bizzotto et al [36], who observed that misinformation can circulate even in expert-led communities when content lacks clarity or nuance. This points to a larger sociotechnical issue that, while professional affiliation may confer perceived authority, it does not automatically guarantee informational accuracy. This has implications for how platforms verify mental health content and for how audiences discern trustworthiness in the absence of explicit medical framing.

The user comments provide an important lens into the psychosocial ecosystem that TikTok favors. Personal testimonials, shared frustrations, and debates around diagnosis indicate that viewers are not merely passive recipients of content but active coconstructors of mental health narratives. The comment threads reflected an informal peer-support infrastructure, albeit one rife with unverified claims and anecdotal medicine. This mirrors observations by Turuba et al [24], who noted that a substantial portion of TikTok mental health content on depression lacked clinical accuracy but retained high personal or emotional resonance. In addition, the study by Steiner-Hofbauer [25], which examined autism portrayal on TikTok (the 50 most-watched for #autism and the 50 most-watched for both #autism and #neurodiversity), revealed that discussions related to ASD, primarily revolved around 3 themes: “people and blogs,” “entertainment,” and “education.” The content tagged only #autism (without “neurodiversity” framing) was overwhelmingly modeled toward entertainment, reaching a significant larger audience (97% of all views in the sample) [25].

By contrast, videos that included the #neurodiversity tag tended to be more educational but were far less popular. Concerningly, the strategy of using a personal experiential framework to portray symptoms in the absence of scientific educational information fuels the spread of misinformation. In addition, in other mental health conditions, SM posts overgeneralize the symptoms of a disorder or overemphasize personal experience as diagnostic evidence [20]. This paradox (emotional engagement vs epistemic reliability) should be at the heart of future digital health interventions. Improving informational quality without losing community relevance remains an important challenge for clinicians, educators, and technology designers.

The present findings should also be interpreted in the context of methodological concerns identified in machine-learning research on deceptive SM activity. In a Prediction Model Risk of Bias Assessment Tool–informed systematic review of 36 machine learning and deep-learning studies, Liu et al [37] found that 86% inadequately addressed class imbalance and highlighted frequent reliance on accuracy despite its limited informativeness in imbalanced datasets. These concerns are particularly relevant to the present study, in which misinformation represented only 21 of 122 (17.2%) videos. Accordingly, the revised model incorporated class weighting and stratified cross-validation and was evaluated using the confusion matrix, recall, *F*_1_-score, balanced accuracy, AUROC, average precision, and a majority-class baseline, with bootstrap CIs. Notably, although the majority-class classifier achieved higher raw accuracy, it identified no misinformation cases, reinforcing the importance of performance metrics that account for minority-class detection. Even with these safeguards, the observed discrimination remained modest, and the model should therefore be regarded as exploratory rather than as a deployable misinformation-detection system.

### Limitations

This study has several limitations. The analytic sample was relatively small and was derived through secondary screening of a previously collected 1000-video dataset rather than through a sampling strategy designed specifically for child and adolescent mental health content. Consequently, the sample may not fully represent the breadth of mental health information encountered by young TikTok users, and estimates for sparsely represented topics should be interpreted cautiously. The final sample also included only English- and Spanish-language videos; although French was eligible in the parent dataset, no French-language videos met the additional child and adolescent mental health criterion. These findings therefore may not generalize to content in other languages or cultural contexts.

The classification of misinformation, disinformation, and authenticity necessarily involved subjective judgment. Although interrater reliability was high for content, intent, and authenticity, creator intent cannot be directly observed from publicly available videos; the distinction between misinformation and disinformation therefore reflects inference from observable content and context rather than verified knowledge of creators’ intentions. This limitation is particularly important for disinformation, which was identified in only 2 videos and was therefore analyzed descriptively rather than modeled.

The predictive analysis should be considered exploratory. Although the revised L2-penalized logistic regression used only platform-observable characteristics and incorporated class weighting, stratified cross-validation, a majority-class comparator, and bootstrap CIs, discrimination remained modest. The limited number of misinformation cases constrains model stability and precludes interpreting individual coefficients as causal effects, robust risk factors, or evidence of a deployable detection algorithm. Because the study was cross-sectional and did not directly measure algorithmic exposure or recommendation processes, associations between engagement metrics and misinformation should not be interpreted as evidence of algorithmic amplification.

The qualitative component was based on 1 illustrative viewer comment per video rather than an exhaustive or systematically ranked sample of comments. The resulting 116-comment corpus therefore reflects purposive selection and cannot be considered representative of the broader discourse surrounding each video or of TikTok users generally. The identified themes should consequently be interpreted as exploratory patterns rather than estimates of theme prevalence. In addition, TikTok content and recommendation systems evolve rapidly, so the observed content landscape may change over time and replication with larger, prospectively collected samples will be important.

### Recommendations for Educational Video Capsules on Child and Adolescent’s Mental Health

#### Overview

Based on the findings of this study, the following suggested recommendations are proposed to improve the quality and reliability of psychoeducational short-form videos addressing child and adolescent mental health on SM platforms like TikTok. These recommendations are intended to support content creators, educators, and mental health professionals in reducing the spread of misinformation. It is important to note that these are not official guidelines, but rather informed suggestions derived from observed patterns in the analyzed content.

#### Prioritize Fact-Based, Referenced Content

Creators should clearly distinguish evidence-based information from personal opinion or lived experience. When making clinical claims, citing reputable scientific sources, clinical guidelines, or established professional organizations can help viewers identify the evidentiary basis of the information presented.

#### Limit Virality-Driven Design That Compromises Accuracy

Creators should avoid sensational titles, exaggerated claims, or emotionally charged framing when these require oversimplifying complex mental health information. Although some engagement metrics were associated with misinformation in exploratory analyses, these findings do not establish that greater engagement causes or amplifies misinformation. Short-form content should therefore aim to remain engaging while preserving clinical accuracy and appropriate nuance.

#### Clearly Identify Professional Credentials

Presenters should transparently disclose their qualifications, when applicable, either in the video, caption, or profile. Although professional status alone was not a robust indicator of information accuracy in this study, clear disclosure of credentials may help viewers assess the source, scope of expertise, and context of the information being presented. Importantly, professional credentials should not be interpreted as a substitute for evidence-based content or appropriate referencing.

#### Use Culturally and Developmentally Attuned Language

Given the adolescent and young adult audience on TikTok, videos should strike a balance between accessibility and scientific rigor. Creators should avoid medical jargon but ensure clinical concepts are not distorted. Short analogies, relatable metaphors, or storytelling can enhance comprehension while preserving accuracy.

#### Encourage Critical Thinking and Caution

Include verbal or written cues that promote media literacy, such as “Talk to a professional before making changes” or “This is general advice and your situation may differ.” Such statements can mitigate overreliance on SM as a diagnostic or treatment source and encourage responsible information-seeking behavior among youths.

### Conclusions

This study provides a mixed methods analysis of child and adolescent mental health content on TikTok and highlights important concerns regarding the accuracy and evidentiary basis of psychoeducational information available on the platform. Misinformation was identified in 21 of 122 (17.2%) videos, whereas disinformation was uncommon (2/122, 1.6%). Misinformation was particularly frequent among videos addressing ASDs and mood disorders, although estimates for less frequently represented topics should be interpreted cautiously because of small denominators. While nearly half of the videos were coded as primarily fact-based, explicit reference to external scientific sources was uncommon, occurring in only 9 of 122 (7.4%) videos.

The exploratory L2-penalized logistic regression indicated that platform-observable characteristics provided only modest discrimination of misinformation. This finding underscores the difficulty of identifying misleading mental health content from surface-level characteristics such as engagement, video duration, topic, hashtags, or disclosed credentials alone and does not support their use as stable or causal risk factors. Qualitative analysis further showed that viewers used comment sections to share personal experiences, discuss diagnosis and misdiagnosis, express frustration with mental health care, provide peer support, and exchange treatment-related experiences.

Taken together, these findings illustrate the dual role of TikTok as both a source of accessible mental health information and a space in which inaccurate or insufficiently sourced claims may circulate alongside meaningful peer interaction. Efforts to improve online mental health information for young people should promote reliable and transparent sourcing, encourage creators to clearly identify their professional credentials and the limitations of the evidence presented, and strengthen developmentally appropriate digital health literacy. These efforts should also preserve the peer support and lived-experience perspectives that make social media engaging and meaningful to young users. Future research using larger and prospectively collected samples is needed to determine which characteristics reliably distinguish accurate from misleading mental health content and how such content influences young users’ knowledge, attitudes, and help-seeking behaviors.

## Abbreviations

ADHD: attention-deficit/hyperactivity disorder
ASD: autism spectrum disorder
AUROC: area under the receiver operating characteristic curve
SM: social media

## Data Availability

The dataset analyzed during this study are available from the corresponding author on reasonable request.
